# Dexmedetomidine Inhibits Hippocampal Neuronal Damage Caused by Persistent Isoflurane-Induced Hypotension in Rat Model of Chronic Cerebral Hypoperfusion

**DOI:** 10.7759/cureus.61522

**Published:** 2024-06-02

**Authors:** Takashi Mino, Shinichi Nakao, Atsuhiro Kitaura, Tatsushige Iwamoto, Seishi Kimura, Yasufumi Nakajima, Tatsuki Itoh, Takao Satou

**Affiliations:** 1 Anesthesiology, Kindai University Faculty of Medicine, Osaka, JPN; 2 Anesthesiology, Perioperative Management Center, Okanami General Hospital, Mie, JPN; 3 Food Science and Nutrition, Kindai University Faculty of Agriculture, Osaka, JPN; 4 Diagnostic Pathology, Kindai University Hospital, Osaka, JPN

**Keywords:** dexmedetomidine, chronic cerebral hypoperfusion, hippocampal neuronal damage, cerebral white matter lesion, hypotension

## Abstract

Purpose

The purpose of this study was to investigate the effect of dexmedetomidine (DEX) on hypotension-induced neuronal damage in a chronic cerebral hypoperfusion (CCH) model of rats, an established model of cerebral white matter lesions (WML) in humans, which is prevalent in the elderly and closely related to cognitive decline.

Methods

The CCH model rats were randomly assigned to one of four groups: normotension + no DEX (NN) group (n = 6), normotension + DEX (ND) group (n = 6), hypotension + no DEX (HN) group (n = 6), or hypotension + DEX (HD) group (n = 6). Under isoflurane anesthesia, mean arterial blood pressure was maintained at or above 80 mmHg (normotension) or below 60 mmHg (hypotension) for a duration of two hours. The DEX groups received 50 μg of DEX intraperitoneally. Two weeks later, the Y-maze test and, after preparing brain slices, immunohistochemical staining were performed using antibodies against neuronal nuclei (NeuN), microtubule-associated protein 2 (MAP2), glial fibrillary acidic protein (GFAP), and Ionized calcium-binding adapter molecule 1 (Iba1).

Results

Behavioral observations showed no significant differences among the groups. Significant reductions of both NeuN-positive cells and the MAP2-positive area were found in the hippocampal CA1 in the HN group compared with NN and ND groups, but not in the HD group. GFAP and Iba-1-positive areas were significantly increased in the HN group, but not in the HD group.

Conclusion

DEX significantly ameliorated hypotension-induced neuronal damage and both astroglial and microglial activation in the CA1 region of CCH rats.

## Introduction

Cerebral white matter lesions (WMLs) are produced by chronic cerebral hypoperfusion (CCH), in the absence of cerebral infarction, which typically occurs when cerebral blood flow (CBF) is reduced to < 20% of normal levels, and in which neuronal cell bodies are mainly damaged [1-4]. The cerebral white matter is composed of myelinated axons and glia, especially oligodendrocytes that form the myelin sheath, whereas the cerebral gray matter is mainly composed of neuronal cell bodies (perikaryon), dendrites, and astroglial cells [4]. WML develops with age, particularly in patients with arterial hypertension, diabetes mellitus (DM), or cardiovascular diseases, is found in 27%-87% of > 65-year-old brains and has been demonstrated to be closely correlated with cognitive decline [1-4]. It has been demonstrated that patients with WML have a marked risk of postoperative cognitive dysfunction (POCD) [5], and the purpose of this study was to investigate whether WML is a major cause of POCD [6].

A rat model of CCH is characterized by WML and cognitive impairment and has been well-established as a model for patients with WML [4,7,8]. In this model, CBF decreases to 40%-82% of normal levels over a prolonged period without cerebral infarctions. CCH per se induces marked microglial activation and axonal damage in some brain regions, such as the caudoputamen, optic tract, corpus callosum, and cerebral cortex, without causing neuronal damage, i.e., cerebral infarction [4,7,8]. There is abundant evidence to suggest that oxidative stress and inflammatory responses play pivotal roles in WML [8,9]. We previously demonstrated that hypocapnia or hypotension, which leads to a further reduction of CBF, caused neuronal damage in the caudoputamen or hippocampus of rats with CCH [10,11], and ketamine, an N-methyl-D aspartate (NMDA) receptor antagonist and anti-inflammatory agent [12], ameliorated this neuronal damage [13]. Dexmedetomidine (DEX), a specific α2 adrenaline receptor agonist and an anti-inflammatory agent [14,15], is often used for sedation during mechanical ventilation in anesthesia and intensive care and has been reported to exhibit neuroprotective effects in not only animals with various insults to the brain but also humans [16,17]. In the present study, we investigated whether DEX could ameliorate neuronal damage induced by hypotension in CCH rats.

## Materials and methods

The study received approval from the animal research committee of Kindai University Faculty of Medicine (KAME-2022-075). Table 1 shows the overview of the groups, including their descriptions and the number of rats in each group. Twenty-four adult male Sprague-Dawley (SD) rats, aged 12 weeks and weighing between 380 and 450 g, were randomly allocated to one of four groups: normotension + no DEX (NN) group (n = 6, 25%), normotension + DEX (ND) group (n = 6, 25%), hypotension + no DEX (HN) group (n = 6, 25%), or hypotension + DEX (HD) group (n = 6, 25%).

**Table 1:** Overview of the groups, their descriptions, and the number of rats in each group.

Group	Description	Number of Tats
NN	normotension + no DEX	6
ND	normotension + DEX	6
HN	hypotension + no DEX	6
HD	hypotension + DEX	6

Preparation of CCH rats

Rats were anesthetized with 4% isoflurane and 50% O_2_ and allowed to respire spontaneously. Before making the skin incision, 0.3 mL of 0.5% lidocaine was administered percutaneously. Bilateral common carotid arteries were then ligated using silk suture material [7,8,10,11]. Sham-operated rats underwent the same surgical procedure but without carotid artery ligation. Following the procedure, rats were maintained under controlled environmental conditions (ambient temperature: 23-26 °C, 12/12-hour light/dark cycle with lights on at 7 AM), with ad libitum access to food and water.

Induction of hypotension

Two weeks following the establishment of the CCH model, rats were anesthetized with isoflurane, intubated with the assistance of 0.1 mg/kg of vecuronium, and mechanically ventilated as follows: initial anesthesia induction was achieved with 4% isoflurane and 100% O_2_ administered via a mask. Subsequently, anesthesia maintenance involved 3% isoflurane and 50% O_2_, while mechanical ventilation was initiated. A tail vein cannulation was performed for drug administration, and the right femoral artery was cannulated to facilitate arterial blood pressure measurement and blood gas analysis sampling.

In the NN and ND groups, mean arterial pressure (MAP) was maintained at or above 80 mmHg (normotension) through phenylephrine injections if required. For the HN and HD groups, experimental conditions mirrored those of the NN and ND groups, but MAP was sustained below 60 mmHg (hypotension) by adjusting the isoflurane concentration. In the ND and HD groups, 50 μg (0.5 mL) of DEX was administered intraperitoneally, while an equal volume of normal saline was administered to the NN and HN groups immediately after mechanical ventilation setup.

Temporalis muscle temperature was monitored and maintained between 36.5 and 37.5°C using a warm water mattress and heating lamp. After maintaining MAP within the target range for two hours, anesthesia was discontinued, and tracheal extubation was performed. Rats were then maintained under the aforementioned conditions for an additional 14 days.

Behavioral test

**Figure 1:**
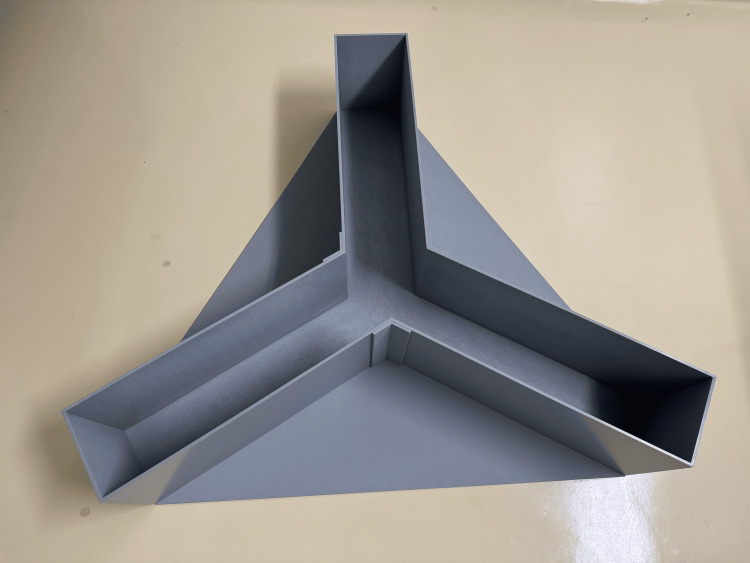
Y-maze

**Figure 2:**
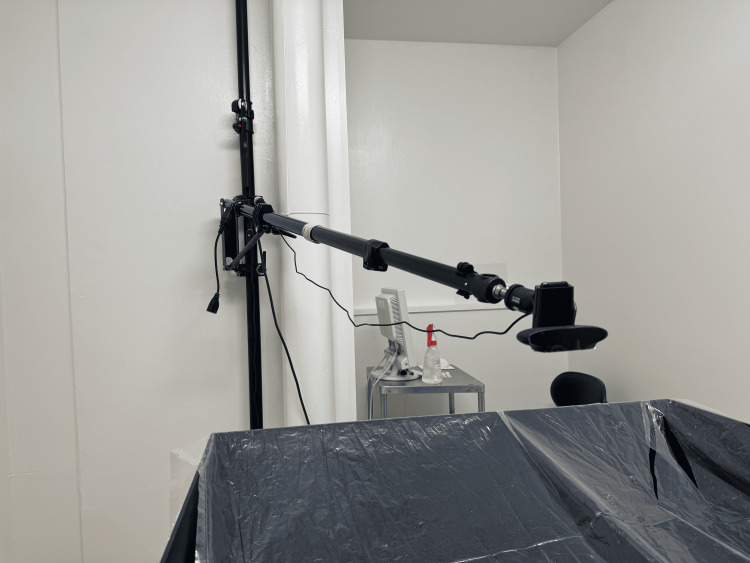
A video camera mounted on the top of the Y-maze

The behavioral test was performed 14 days after rats were exposed to hypo- or normotension by isoflurane. Rats underwent a spontaneous alternation test to measure working memory in the Y-maze (Figure 1). They were divided into three 120-degree-angle arms (30 cm high, 50 cm long, and 15 cm wide) labeled A, B, and C. The equilateral triangle formed by the three arms was 15 cm long. Arms were randomly set up as the start arm, the novel arm, and the other arm. Each rat was placed in the start arm. A video camera mounted on the top of the Y-maze recorded their movements for 8 min (Figure 2), and the percentage of alternations (entries into arms different from the previous two entries) as spatial memory was calculated using the following formula (Alternations/Arm Entries - 2) x 100. After the behavioral test of each rat, the Y-maze was cleaned to remove odors and residues so that they would not affect the test results. During the behavioral test, the investigator was not visible to the rats. Sham-operated rats were set up for behavioral tests only.

Preparation of brain slices and histopathology

Fourteen days following the completion of the behavioral test, rats were deeply anesthetized with isoflurane and transcardially perfused with 0.001 mol/L phosphate-buffered saline, followed by a fixative solution composed of 4% paraformaldehyde and 0.2% picric acid in 0.1 mol/L phosphate buffer (pH 7.4). The brain was then extracted, post-fixed in 4% paraformaldehyde in 0.1 mol/L phosphate buffer (pH 7.4) for 24 hours, and subsequently stored in 20% sucrose solution in 0.1 mol/L phosphate buffer (pH 7.4).

Brain tissue sections were obtained using a cryostat (thickness: 20 μm), and these sections were utilized for histological, immunohistochemical, or immunofluorescent analyses. Unless otherwise specified, all incubation procedures were conducted at room temperature.

Brain sections were treated with 3% H_2_O_2_ in Tris-buffered saline (TBS: 0.1 M Tris-HCl, pH 7.5, 0.15 M NaCl) containing 0.1% TritonX-100 (TBS-T) for 30 minutes. The sections were then washed three times with TBS-T, and incubated overnight at room temperature with mouse monoclonal antibodies against neuronal nuclei (NeuN) (Millipore, Billerica, MA, USA), as a neuronal marker that recognizes nuclear proteins, microtubule-associated protein-2 (MAP-2) (Sigma Aldrich, Burlington, MA, USA), which is located almost exclusively in the neuronal perikaryal and dendrites and the reduction of which indicates neuronal damage, and ionized calcium-binding adaptor molecule 1 (Iba1) (Wako Pure Chemical, Osaka, Japan), as a marker of microglia, or rabbit polyclonal antibody against glial fibrillary acidic protein (GFAP) (DAKO, Glostrup, Denmark), as a marker of astrocytes. After thorough washing, the sections were further incubated with HISTIFINE Rat-PO (multi)-kit (Nichirei, Osaka, Japan) consisting of a mixture of peroxidase-conjugated anti-mouse and rabbit IgG as a secondary antibody for 60 min at room temperature. The HISTIFINE Rat-PO kit contains pre-absorbed rat serum, and nonspecific binding by rat serum in damaged rat tissue was negligible. The label was visualized for 5 min using diaminobenzidine (DAB; Vector Peroxidase Substrate Kit; Vector Laboratories, Burlingame, CA, USA) and contrast-stained with hematoxylin, except for sections incubated with NeuN antibody. Hematoxylin-contrast staining was performed on all sections except for those incubated with NeuN antibody.

Morphometry and statistical analyses

Neuronal damage was quantified by counting the number of morphologically normal cell nuclei stained with NeuN in a given 0.04-mm^2^area of the hippocampal CA1 region in each hemisphere. Normal cell bodies and dendrites were calculated using image-processing software (Olympus BX53, Tokyo, Japan) for MAP2-positive areas in a given 0.04-mm^2^ area of the hippocampal CA1 region in each hemisphere. Based on previous experiments [11], the hypothesis of this study was that there was a 30% difference between HN and HD groups in the development of neuronal damage. To detect this difference with a power of 80% and a P-value of 0.05, power calculations indicated that a sample size of at least six animals per group was required. Sample sizes were calculated using the G*power 3.1 statistical power analysis program (Heinrich-Heine-University, Düsseldorf, Germany). Statistical comparisons of physiological variables between groups were performed using one-way analysis of variance (ANOVA) followed by the Bonferroni post-hoc test, and histological data between groups were analyzed using Kruskal-Wallis statistics followed by Dunn's post-hoc test. Results are expressed as mean ± standard deviation (SD) or median (IQR: interquartile range). p-values < 0.05 were considered significant. The power of statistical comparison was assessed using JMP14.2 Statistical Discovery Software (SAS Inst., Inc., Cary, NC).

## Results

Table 2 shows the physiological parameters observed during mechanical ventilation. MAP exhibited a significant decrease in the HN and HD groups compared to the NN and ND groups. Heart rate, pH, PaO_2_, PaCO_2_, temperature, and blood glucose levels were all within normal physiological ranges and showed no significant differences across the groups.

**Table 2:** Physiological variables during anesthesia. Values are mean ± SD (standard deviation) NN group: normotension + no DEX group, ND group: normotension + DEX group, HN group: hypotension + no DEX group, HD group: hypotension + DEX group *P < 0.05 vs. NN and ND groups

	NN group	ND group	HN group	HD group
Mean arterial pressure (mmHg)	108 ± 10	112 ± 7	55 ± 4*	56 ± 3*
Heart rate (bpm)	220 ± 40	238 ± 38	253 ± 34	241 ± 23
pH	7.422 ± 0.09	7.403 ± 0.07	7.392 ± 0.11	7.42 ± 0.03
PaCO_2 _(mmHg)	42.2 ± 4.6	40.5 ± 3.8	39.4 ± 4.4	43.1 ± 3.1
PaO_2 _(mmHg)	180.6 ± 49.3	201.3 ± 60.1	199.2 ± 52.5	210.4 ± 33.7
Temperature (℃)	37.0 ± 0.2	37.1 ± 0.2	37.1± 0.1	37.1 ± 0.1
Glucose (mg/dL)	150 ± 37	139 ± 28	150 ± 41	130 ± 23

**Figure 3:**
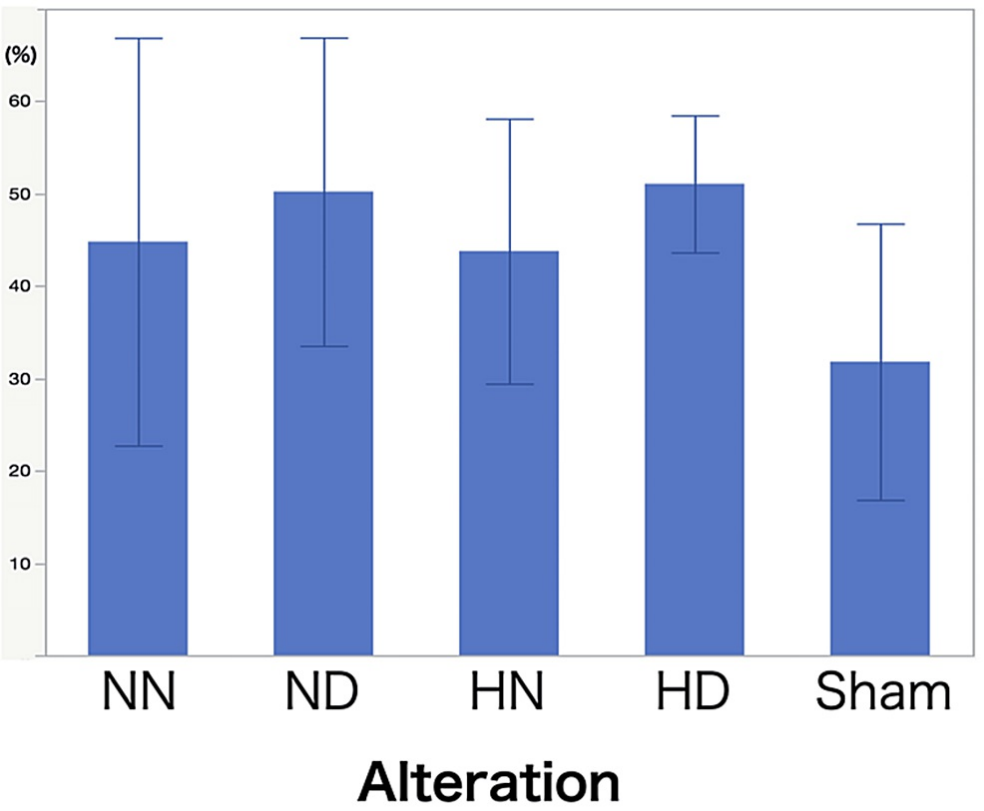
Behavioral test. Results of a spontaneous alternation test to measure working memory in the Y-maze 14 days after rats were exposed to isoflurane-induced hypotension or normotension. In the behavioral testing, evaluation was conducted using alteration scores (%). The results are shown as mean and standard deviation. There were no significant differences in the alteration scores among the four groups of rats: NN (normotension + no dexmedetomidine) group, ND (normotension + dexmedetomidine) group, HN (hypotension + no dexmedetomidine) group, and HD (hypotension + dexmedetomidine) group. Behavioral testing showed no significant differences among the groups. Values are mean ± SD (standard deviation) *P < 0.05 vs. NN and ND groups

**Figure 4:**
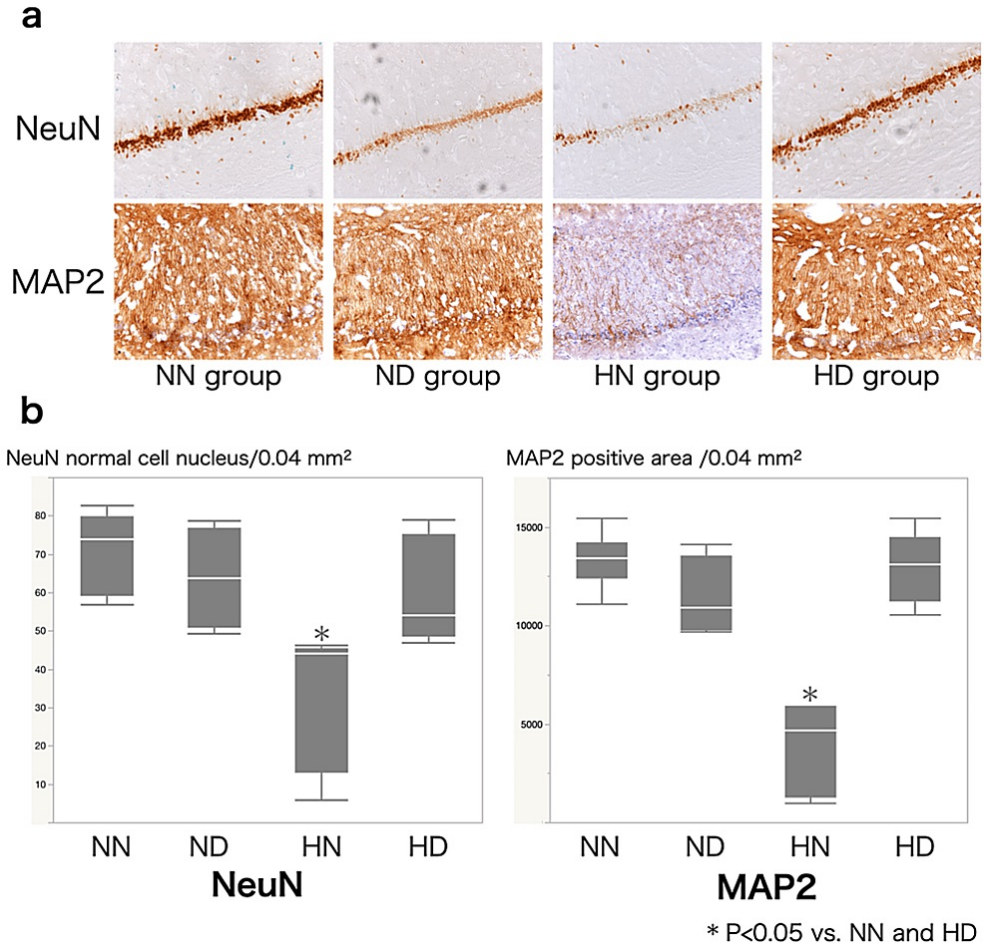
Photomicrograph of both NeuN and MAP2 in the hippocampal CA1 region. (a) Photomicrograph of both NeuN and MAP2 in the hippocampal CA1 region in the NN (normotension + no dexmedetomidine) group, ND (normotension + dexmedetomidine) group, HN (hypotension + no dexmedetomidine) group, and HD (hypotension + dexmedetomidine) group. (b) Box and whisker plots depicting the counts of normal neuronal cells in predefined areas (0.04 mm2) within the hippocampal CA1 region across the NN, ND, HN, and HD groups. The values are presented as the median (represented by the transverse line inside the boxes), interquartile range (IQR) denoted by the boxes, and the 10th–90th percentiles illustrated by the whiskers. NN and ND groups did not show a reduction in normal cell counts, but they were significantly decreased in the HN group. The HN group showed a significant reduction in the MAP2-positive area of the hippocampal CA1 region, but dexmedetomidine pretreatment protected against the reduction. *P < 0.05 vs. NN and HD groups

**Figure 5:**
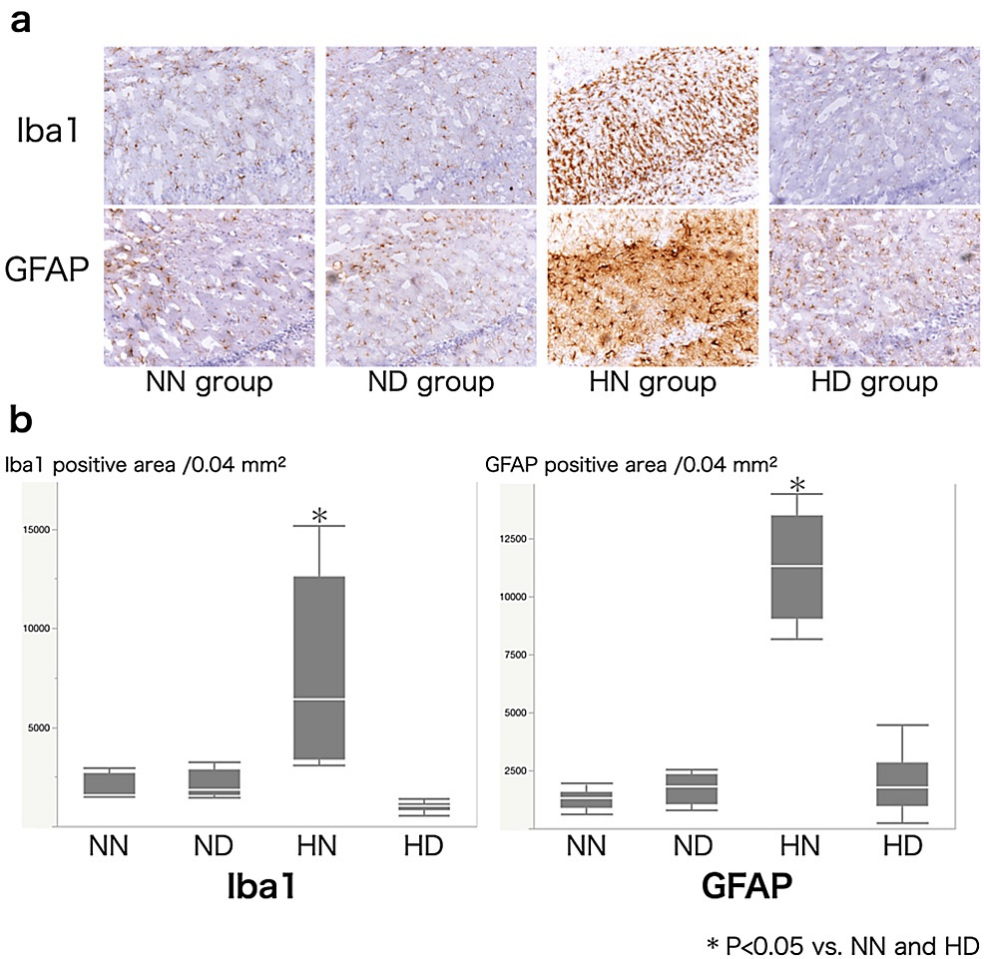
Photomicrograph of both Iba1 and GFAP in the hippocampal CA1 region. a Photomicrographs of both Iba1 and GFAP immunohistochemical staining of the hippocampal CA1 region in the NN (normotension + dexmedetomidine non-administration) group, ND (normotension + dexmedetomidine) group, HN (hypotensive + dexmedetomidine non-administration) group, and HD (hypotensive + dexmedetomidine) group. b Box and whisker plots of Iba1 and GFAP-positive regions in predetermined areas (0.04 mm2) of the hippocampal CA1 region in NN, ND, HN, and HD groups. NN and ND groups did not show an increase in the positive area, but it was significantly increased in the HN group. Values are expressed as median (transverse line inside boxes), IQR (boxes), and 10–90 percentiles (whiskers). *P < 0.05 vs. NN and HD groups

Behavioral testing showed no significant differences among the groups (Figure 3). Figure 4a shows a representative photomicrograph of immunostaining of both NeuN and MAP2 in the hippocampal CA1 region. CCH per se did not reduce normal cell counts (Figures 4a, 4b; NN and ND groups), but they were significantly decreased in the HN group, and DEX pretreatment significantly ameliorated the reduction (HD group). The HN group showed a significant reduction in the MAP2-positive area of the hippocampal CA1 region, but DEX pretreatment protected against this reduction (Figures 4a, 4b). Both microglia and astroglia (Figures 5a, 5b) were significantly activated in the HN group, but DEX significantly suppressed those activations. The morphological change of microglia from ameboid to ramified form was noted in the HN group.

## Discussion

We demonstrated that CCH per se induced WML but not CA1 neuronal damage in rats, and exposure of CCH rats to persistent hypotension resulted in activation of both microglia and astrocytes and CA1 neuronal damage, being consistent with previous studies [7,8,10,11]. Curiously, the results of the behavioral test (Y maze test) did not differ among groups, even with the massive CA1 neuronal damage group (HN group). DEX significantly suppressed the glial activation and neuronal damage in CA1.

WML is caused by CCH without apparent neuronal damage, i.e., cerebral infarction, and is closely associated with cognitive decline [9,10,12-14]. A rat model of CCH has been used as a well-established model of global WML and vascular dementia in humans [9,10,12-14]. We previously demonstrated that hypocapnia [10] or hypotension [11], both of which can reduce CBF, caused massive CA1 neuronal damage as well as marked microglial and astroglial activation in CCH rats, but those insults per se could not cause the neuronal damage in normal rats. In the present study, we confirmed that CCH per se did not induce pyramidal neuronal cell damage in CA1; persistent hypotension caused the neuronal damage (Figures 2a, 2b), probably because CBF in CA1 of CCH rats was reduced to less than 20% of normal blood flow.

The definition of hypotension in rats is not clear, but as normal mean blood pressure under general anesthesia is around 90 mmHg [10,18] and the mean blood pressure drops to 40 mmHg during hemorrhagic shock in SD rats, we defined normotension as ≥ 80 mmHg and moderate hypotension as ≤ 60 mmHg [18].

NeuN protein is localized in nuclei and perinuclear cytoplasm of most neurons in the central nervous system of mammals and used as a neuromarker [19], and MAP2, which stabilizes microtubules, is located almost exclusively in perikaryal regions and dendrites. Therefore, a decrease in the count of NeuN staining or the area of MAP2 staining indicates neuronal damage [10]. Microglia are immune cells that are resident in the brain and activated in response to ischemia and other insults, secreting various factors such as cytokines, chemokines, and oxygen radicals, and activated microglia induce neuronal damage [10]. In this study, not only an increase in the number of microglia but also a morphological change of microglia from ameboid to ramified form was noted in the HN group, indicating that further reduction in the CBF by persistent hypotension caused the glial activation, which was significantly suppressed by DEX in the HD group (Figures 3a, 3b). Astrocytes are heterogeneous and have diverse functions that can contribute to tissue repair and promote CNS pathology in the context of trauma, infection, and neurodegenerative diseases [20]. Astrocytes are activated by inflammation and also contribute to the progression of inflammation.

CA1 of the hippocampus is highly vulnerable to global cerebral ischemia not only in animals but also in humans [21,22], and the damage of CA1 pyramidal cells causes cognitive damage, especially special learning impairment, which can be detected by Morris water maze testing in rodents [21,23,24]. In the present study, however, rats whose CA1 neurons were markedly damaged did not show memory impairment on Y maze testing. The discrepancy may be due to the sensitivity of the Y maze test and/or the results of some reports which showed that the intensity of CA1 neuronal damage did not necessarily correlate with the severity of behavioral impairment [21,23,24].

DEX is a specific α2 adrenaline receptor agonist and has been demonstrated to exhibit anti-inflammatory activity in animals [14] and humans [14,15], and consequently provide cardio- [15] or neuro- [16,17] protection against various insults. In the present study, DEX significantly suppressed the activation of both microglia and astrocytes, probably through its anti-inflammatory effect, and exerted neuronal protection in CA1 in CCH rats exposed to persistent hypotension. Although DEX is usually used as a continuous infusion in clinical settings, in the present study, a single dose of 50 µg DEX was administered intraperitoneally to rats. However, it has been reported that a single administration of intraperitoneal DEX could induce prolonged DEX plasma elevation and exhibit various stable effects in rats: Kato et al. reported that the sedative effect was prolonged over 90 min after 30 or 100 µg/kg DEX intraperitoneal administration in rats [25]. Dawson and colleagues demonstrated that the antinociceptive effective dose 50 (ED50) of a single intraperitoneal administration of DEX in rats was 27.6 ± 5.1 µg/kg, and the antinociceptive effect persisted for up to 90 min [26]. Bol et al. reported that plasma DEX levels remained high even at 90 min after intraperitoneal administration of 30 µg/kg DEX to rats [27].

CCH rats feature WML without apparent neuronal damage, i.e., cerebral infarction. WML is prevalent in elderly people and patients with DM, hypertension, and cerebrovascular diseases [28-30]. In an intensive care unit, patients under mechanical ventilation are sometimes elderly have hypertension, DM, and/or cerebrovascular diseases, and are at risk of persistent hypotension [31-33]. As DEX is widely used for sedation in patients under mechanical ventilation, we believe that it is a good choice for sedation, especially in patients with WML from the perspective of neuroprotection.

Our study had several limitations. First, we did not measure the actual CBF of rats; thus, we did not know the exact reduction of CBF caused by hypotension. Second, we used isoflurane as a hypotension-inducing agent because we wanted to avoid using another drug to induce hypotension. General anesthetics, including isoflurane, theoretically have some neuroprotective effects on the brain [34,35], but in contrast, only isoflurane has been reported to have some harmful effects on the nervous system [36]. In the present study, however, as all groups were exposed to isoflurane, we believe that the effects of isoflurane could be disregarded.

## Conclusions

In a rat model of CCH, persistent hypotension caused inflammation in the brain probably due to CBF reduction and consequently hippocampal CA1 neuronal damage, which all were suppressed by DEX. The results suggest that DEX is a good choice for sedation of patients under mechanical ventilation, especially elderly patients, and/or patients with hypertension, DM, and/or cerebrovascular diseases to prevent neuronal damage and/or cognitive decline.
